# Inference of trajectory presence by tree dimension and subset specificity by subtree cover

**DOI:** 10.1371/journal.pcbi.1009829

**Published:** 2022-02-08

**Authors:** Lovemore Tenha, Mingzhou Song

**Affiliations:** 1 Department of Computer Science, New Mexico State University, Las Cruces, New Mexico, United States of America; 2 Graduate Program in Molecular Biology and Interdisciplinary Life Sciences, New Mexico State University, Las Cruces, New Mexico, United States of America; University of California San Diego, UNITED STATES

## Abstract

The complexity of biological processes such as cell differentiation is reflected in dynamic transitions between cellular states. Trajectory inference arranges the states into a progression using methodologies propelled by single-cell biology. However, current methods, all returning a best trajectory, do not adequately assess statistical significance of noisy patterns, leading to uncertainty in inferred trajectories. We introduce a tree dimension test for trajectory presence in multivariate data by a dimension measure of Euclidean minimum spanning tree, a test statistic, and a null distribution. Computable in linear time to tree size, the tree dimension measure summarizes the extent of branching more effectively than globally insensitive number of leaves or tree diameter indifferent to secondary branches. The test statistic quantifies trajectory presence and its null distribution is estimated under the null hypothesis of no trajectory in data. On simulated and real single-cell datasets, the test outperformed the intuitive number of leaves and tree diameter statistics. Next, we developed a measure for the tissue specificity of the dynamics of a subset, based on the minimum subtree cover of the subset in a minimum spanning tree. We found that tissue specificity of pathway gene expression dynamics is conserved in human and mouse development: several signal transduction pathways including calcium and Wnt signaling are most tissue specific, while genetic information processing pathways such as ribosome and mismatch repair are least so. Neither the tree dimension test nor the subset specificity measure has any user parameter to tune. Our work opens a window to prioritize cellular dynamics and pathways in development and other multivariate dynamical systems.

## Author summary

Modern biology now routinely studies transcriptome profiles during development. This practice demands computational methods to quantify dynamical changes in cellular states and their heterogeneity. Many methods process single-cell transcriptome data to reconstruct cellular trajectories, which are orderings of cells as they progress from an early to a late developmental stage. Due to noise in transcriptome data, there is a great need to quantify how likely observed data present a trajectory-like pattern due to chance. To address this need, we developed a tree dimension test to quantify evidence for trajectory presence in multivariate data based on graph-theoretical concepts. By this test, one may reject trajectory presence due to low data quality, or accept a trajectory with high statistical significance. Now one can rank biological pathways by their trajectory quality. We also introduce a subset specificity measure to quantify how cellular or pathway dynamics are tissue specific. We found that pathway tissue specificity is highly conserved between human and mouse. Trajectory presence testing and subset specificity offer a unique informatics tool set to study developmental biology.

## Introduction

Recognizing dynamic transitions between cellular states can generate deeper understandings of development, disease processes, or environmental response inside a biological system. Single-cell biology has facilitated the exploration of such dynamics. Trajectory inference harnesses omic data at cellular resolution to identify cellular state progressions, for which many computational methods have been developed [1, 2, 3]. Extensive evaluation shows that many methods can infer certain types of trajectory with a high degree of accuracy without the use of prior biological information [4]. Trajectory inference methods, often operating on a low dimensional manifold embedded in a high dimensional space, employ various strategies to capture a trajectory. Methods such as TSCAN make use of minimum spanning trees (MSTs) built on cluster centroids to capture a trajectory structure underlying the data [5]. SLICER uses locally linear embedding and *k*-nearest neighbor graphs to find a trajectory [6]. Topological data analysis finds compact representation of complex high dimensional data [7, 8]. Vandaele et al developed a method for inferring topological structures in graph data, applicable to trajectory inference [9]. They highlighted some challenges [10]: most methods tend to underestimate the number of leaves in graph-representations of trajectories. They also showed that topological information correlates to the performance of consecutive cell trajectory inference algorithms and many datasets with trajectory lack sufficient topological information for effective inference.

To our knowledge, however, existing methods mostly assume trajectory presence in the data and therefore always infer a trajectory regardless of statistical significance. For technical or biological reasons, an experiment may not capture a trajectory pattern even if one is expected. A best trajectory might have caught only random variations arising by chance. Further, no method is available to prioritize trajectory presence in subspace spanned by genes on biological pathways, whose dynamics can be quite distinct from cellular trajectories involving all genes. Lastly, no known statistics have been applied to measure the tissue specificity of cellular or pathway dynamics. Such limitations seriously hamper our capacity to interpret salient signals hidden in multivariate biological data.

To address the trajectory presence problem, we establish a tree dimension test (TDT) that exploits graph-theoretic statistics to characterize patterns indicating the existence and specificity of dynamical processes in observed data. Our method premises that the presence of trajectory patterns in multivariate data can be measured by the degree of linearity and branching in the corresponding Euclidean MST (EMST) of the original data. We introduce a statistical framework based on the tree dimension measure *T*_*d*_ of EMST to accomplish the task. It calculates a test statistic *S* derived from tree dimension measure *T*_*d*_ to quantify statistical evidence for trajectory presence, using a null distribution that is log-normal over the population of spherical multivariate normal random vectors.

To study the specificity of trajectory dynamics in a context such as tissue type, we introduce a subset specificity measure, which is based on the minimum subtree cover of a given subset. Tissue specificity measures the differential expression of pathways in different developing tissue types, thereby enabling pathway behavior characterization.

We evaluated our method on both simulated and real single-cell data, where TDT substantially outperforms tree diameter and number of leaves in recognizing presence of trajectory patterns in the data. We applied our method to test the presence of pathway trajectories in transcriptome data of developing mammalian tissue types and then quantified the tissue specificity of pathway dynamics. Indeed, overall cellular trajectories are qualitatively distinct from specific pathway trajectories during development. Several signal transduction pathways showed high tissue specificity, while low tissue specificity is observed for genetic information processing pathways during mammalian development.

Despite substantial work in graph and topology characterization of multivariate data, our method adds a unique and rigorous statistical perspective to the analysis of dynamical patterns. Since our method addresses the more general question regarding presence of trajectory, it does not require the identification of specific trajectories, and can therefore, be used upstream of a trajectory finding method. Our biological findings support the usefulness of the tree-dimension framework, applicable to molecular biological data with increasingly complex dynamics due to modern biotechnology innovations that steadily improve temporal and cellular resolutions.

## Methods

**Fig 1:**
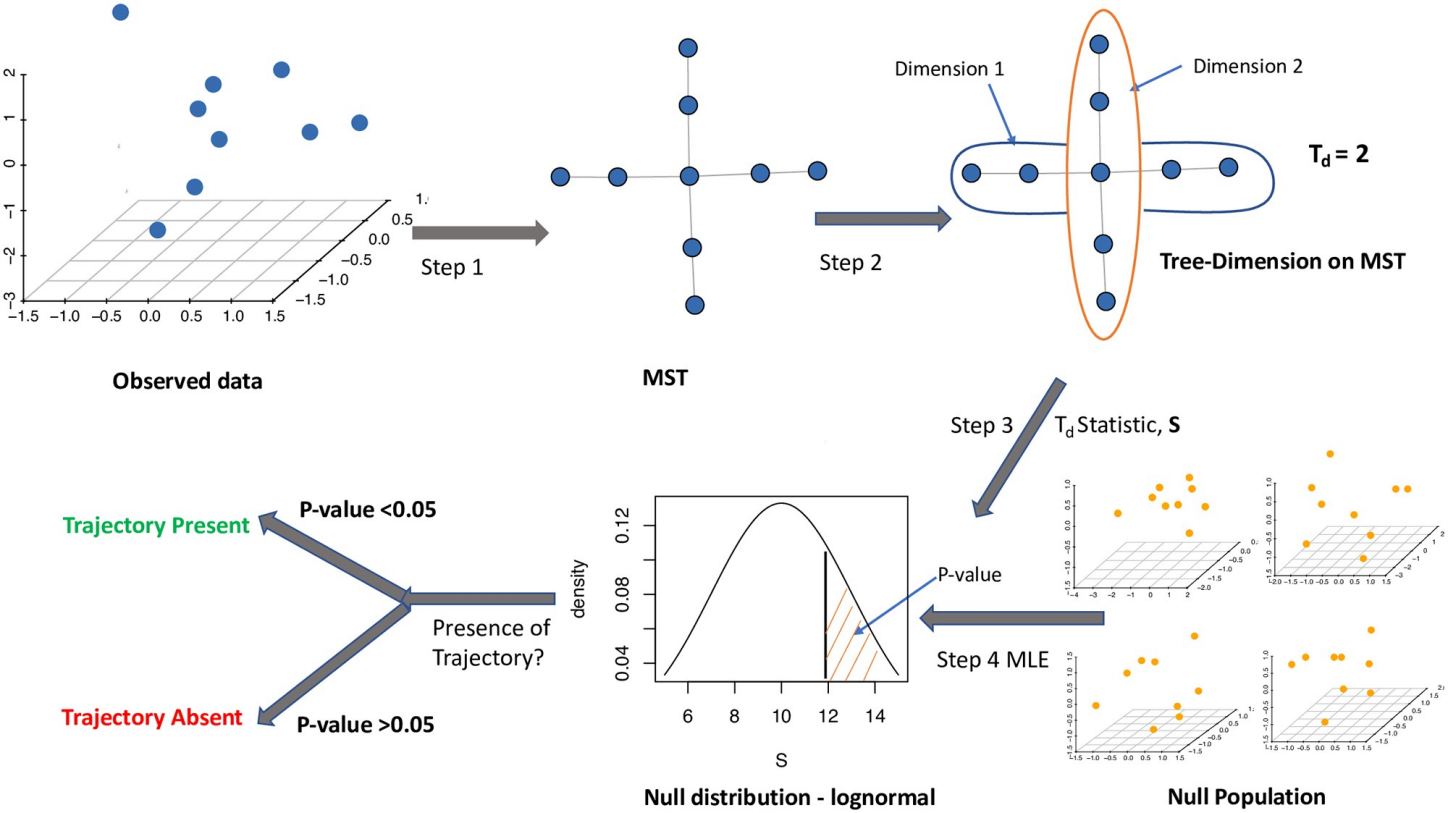
Overview of the tree dimension test for trajectory presence. The input is multivariate data. A Euclidean minimum spanning tree (EMST) is computed on the data points. Trajectory inferential test statistic *S* is computed from tree dimension measure *T*_*d*_ of the EMST. A log-normal null distribution of *S* is derived from the null population following a spherical multivariate normal distribution with no trajectory patterns. A *p*-value of the observed statistic *S* is computed to quantify statistical significance for the presence of a trajectory pattern.

We design a tree dimension test (TDT) for trajectory presence in multivariate data. The test has four main steps as summarized in Fig 1. The first step finds an EMST of the input data in a compact form while preserving the underlying signal, if any. The samples in the data are considered vertices of a complete graph, with edges between every pair of vertices. The length of an edge is the Euclidean distance between its two vertices. From the complete graph, an EMST is computed as a compact representation of the data. The second step exploits the EMST representation to test presence of trajectory patterns in the data. The goal was to identify characteristics of an EMST to indicate whether the underlying pattern is dynamically forming a trajectory or simply random. Thus, we have developed tree dimension measure *T*_*d*_ to map an EMST structure to either the presence or absence of trajectory patterns. *T*_*d*_, summarizing the degree of branching of the EMST, is high for a heavily branched EMST, while a low *T*_*d*_ suggests a strong trajectory pattern. Via the third step, a test statistic *S* is calculated based on *T*_*d*_ to integrate statistical support. In the final step, we derive a log-normal null distribution for *S* on a null population of EMSTs from spherical multivariate normal random data. Using the upper-tail probability of the null distribution, we finally compute the statistical significance of *S* to determine trajectory presence.

Two other graph-theoretic statistics are intuitive for characterizing the degree of linearity of a tree. The first is the number of leaves *L* in a tree. A leaf is a vertex with exactly one incident edge in a tree. *L* measures frequencies of branching events in a tree. *L* is minimized if a tree is completely linear, also known as a path graph. On the other end of the spectrum, *L* is maximized on a star tree. However, *L*, heavily subject to noise, tends to downgrade a globally strong trajectory pattern that may have noisy small branches locally. The second is tree diameter *D*_*m*_, the greatest path length between any pair of vertices in a tree. *D*_*m*_ is maximized if a tree is linear and minimized on a star tree, which is maximally branched. *D*_*m*_, effective in recognizing the main trunk of a tree, is insensitive to secondary branches that can represent multifurcating events in a data collection.

### Tree dimension and its measure

We now introduce a tree dimension measure to combine strengths of tree diameter *D*_*m*_ and number of leaves *L* to robustly characterize the degree of branching of a tree. The rationale is to capture the global trajectory pattern while insensitive to local noisy branching in a tree.

**Definition 1** (Tree dimension). We define the first dimension of a tree as one of its longest paths. The (*k* + 1)-th (*k* > 0) dimension of a tree is the (*k* + 1)-th longest path between two leaves not lying on dimensions 1 to *k*. If only one such leaf is available, dimension *k* + 1 is spanned by the shortest path that connects the only leaf to a vertex in a previous dimension. If no such leaf is available, the tree has exactly *k* dimensions. The intermediary vertices of any dimension, however, may lie on a previous dimension.

It is apparent that each dimension of a tree can differ in length, defined by one plus the number of vertices on that dimension but not on a lower dimension.

**Definition 2** (Tree dimension measure). The tree dimension measure *T*_*d*_, quantifying the degree of branching in a tree, is the sum of lengths of each dimension normalized by the length (*D*_*m*_ + 1) of the first dimension. The definition is intuitive but inconvenient to compute. It is not difficult to show that *T*_*d*_ is equivalently determined by the number of vertices *N* in a tree, its diameter *D*_*m*_ and number of leaves *L* by

$$\begin{array}{c} T_{d}=1+\frac{(N-D_{m}-1)+\left\lceil L/2-1\right\rceil }{D_{m}+1}=\frac{N+\left\lceil L/2-1\right\rceil }{D_{m}+1} \end{array}$$ (1)

For the degenerate case of a singleton tree with *N* = 1, we define number of leaves *L* to be one and tree diameter *D*_*m*_ to be zero. This sets *T*_*d*_ = 1 for singleton trees.

**Fig 2:**
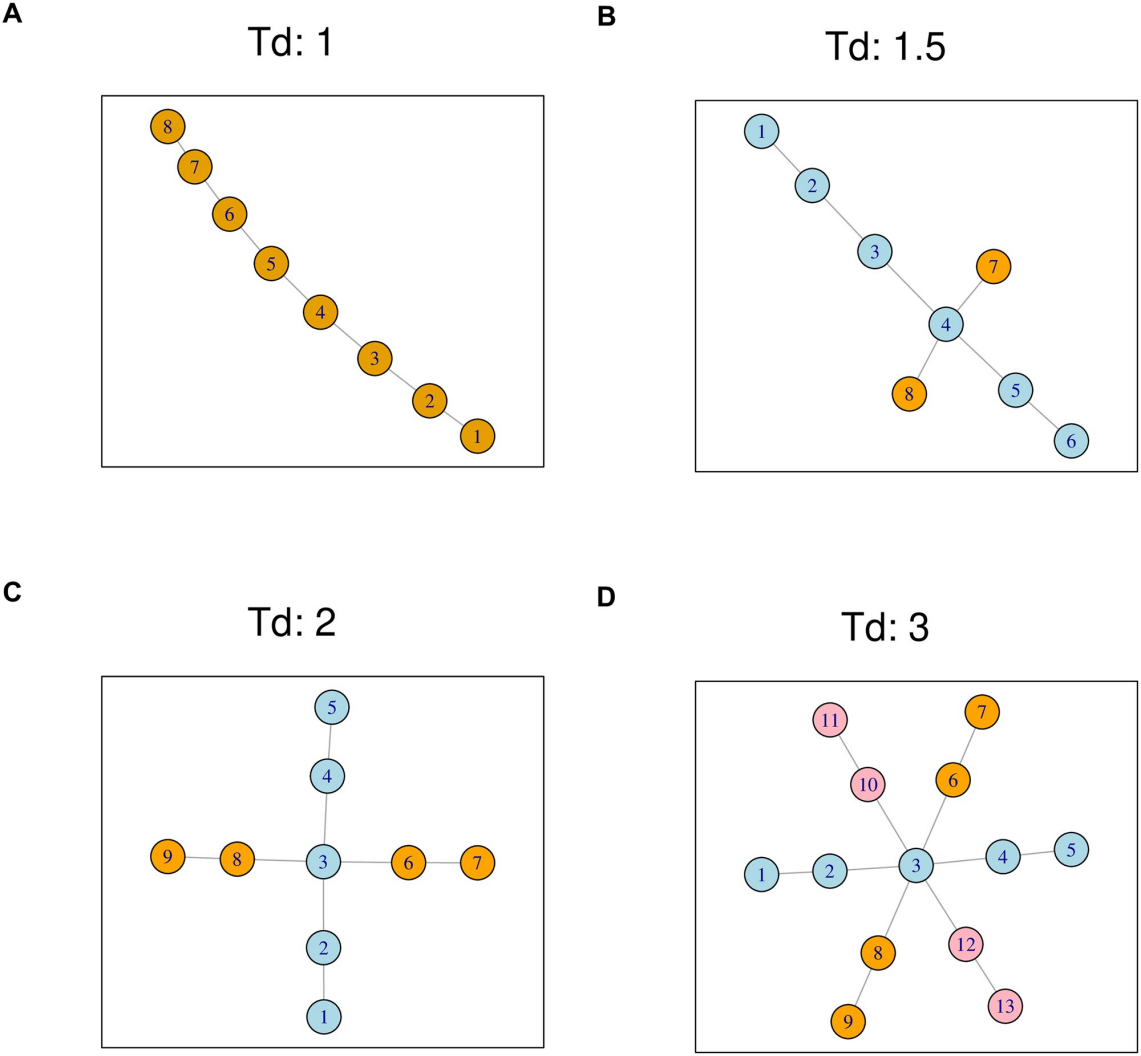
Trees, dimensions, and dimension measures. Each tree dimension is highlighted by a different color. (A) A one-dimensional tree, or a path graph, with tree dimension measure *T*_*d*_ = 1. (B) A two-dimensional tree with *T*_*d*_ = 1.5. (C) A two-dimensional tree with *T*_*d*_ = 2. (D) A three-dimensional tree with *T*_*d*_ = 3.

Fig 2A shows a path graph of dimension one. Both Fig 2B and 2C have two dimensions. The tree in Fig 2D has three dimensions. Their corresponding tree dimension measure *T*_*d*_ are the same with the number of dimensions, except that in Fig 2B *T*_*d*_ is 1.5 as the two dimensions have different lengths.

Algorithm 1 Tree-Dimension-Measure(*X*) calculates tree dimension measure *T*_*d*_ on a multivariate input data *X*. After first obtaining an EMST *T* on *X*, it computes the tree diameter, *D*_*m*_. We apply breadth-first search (BFS) on *T* starting from any vertex, *v* in the tree, while keeping track of the distance of every vertex from *v*. After BFS completion, we select vertex *w*, that has the longest distance from *v*. We run the BFS algorithm again, starting with vertex *w*, while also keeping track of the distance of every vertex from *w*. After completion of BFS, vertex *z* has the longest distance from *w* and the distance of *z* from *w* is the *D*_*m*_. We obtain number of leaves *L* by the number of vertices with degree 1. *D*_*m*_ and *L* are then used to compute *T*_*d*_.

**Algorithm 1** Tree-Dimension-Measure(*X*)

 Input: multivariate data *X*

1 T = Find Euclidean MST on *X*

2 // Find a most distant vertex *w* from some vertex *v* in the tree

 // by breadth-first-search:

 *w* = Breadth-First-Search(*T*, *v*)

3 // Find a most distant vertex *z* from vertex *w* in the tree

 // by breadth-first-search:

 *z* = Breadth-First-Search(*T*, *w*)

4 Tree diameter *D*_*m*_ = the length of the path from *w* to *z*

5 *L* = Number-of-Leaves(*T*)

6 Calculate tree dimension measure *T*_*d*_ by Eq (1)

7 **return** *T*_*d*_

### Mathematical properties of tree dimension measure

**Proposition 1**. *Tree dimension measure T*_*d*_ *is minimized to 1 if and only if a tree is a path/linear graph*.

*Proof*. We consider a tree of one vertex (*N* = 1) a path graph, which always has *T*_*d*_ = 1 by definition. A path graph of *N* ≥ 2 vertices has exactly two leaves (*L* = 2) and all others vertices are of degree two. Tree diameter *D*_*m*_ is thus *N* − 1. Therefore, *T*_*d*_ = 1 by Eq (1).

A non-path graph has *L* > 2. It must follow that ⌈*L*/2 − 1⌉ > 0. As the diameter *D*_*m*_ is less than the number of vertices *N* in any tree, *N* − *D*_*m*_ − 1 ≥ 0. Therefore, we arrive at *N* − *D*_*m*_ − 1 + ⌈*L*/2 − 1⌉ > 0, implying *T*_*d*_ > 1 by Eq (1).

Therefore, *T*_*d*_ is minimized to 1 if and only if the tree is a path graph.

**Proposition 2**. *Tree dimension measure T*_*d*_ *is maximized to* $(N+\left\lceil \frac{N-1}{2}-1\right\rceil )/3$ *if and only if we have a star tree, among all trees of N* ≥ 3 *vertices*.

*Proof*. A star tree of *N* ≥ 3 vertices has *N* − 1 leaves and one vertex of degree *N* − 1. Thus, tree diameter *D*_*m*_ is 2. Therefore, $T_{d}=\left(N+\left\lceil \frac{N-1}{2}-1\right\rceil \right)/3$.

A non-star tree of *N* ≥ 3 must have a diameter *x* > 2 by definition. Let *y* be the number of leaves. As any tree of *N* ≥ 3 vertices has at most *N* − 1 leaves, we have $\left\lceil \frac{y}{2}-1\right\rceil \leq \left\lceil \frac{N-1}{2}-1\right\rceil$. Tree diameter *x* > 2 implies *x* + 1 > 3. Thus, it follows by Eq (1) that $T_{d}=\left(N+\left\lceil \frac{y}{2}-1\right\rceil \right)/\left(x+1\right)<(N+\left\lceil \frac{N-1}{2}-1\right\rceil )/3$.

Therefore, *T*_*d*_ is maximized if and only if we have a star tree, among all trees of *N* ≥ 3 vertices.

### Examples of tree dimension measure, diameter and number of leaves

**Fig 3:**
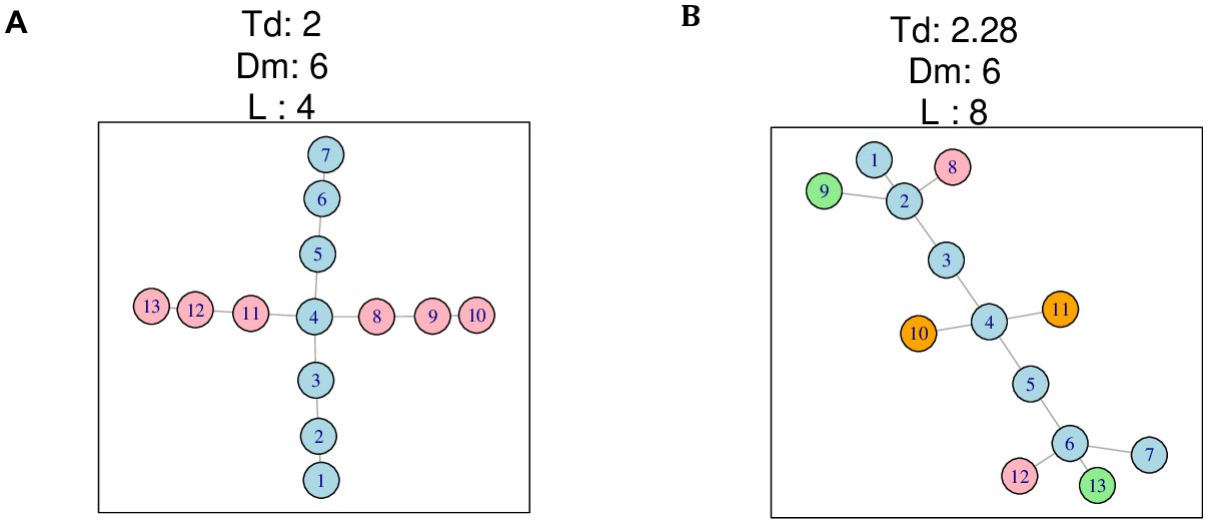
Two trees of the same number of vertices and their tree dimension measure *T*_*d*_, diameter *D*_*m*_ and number of leaves *L*. The color of a vertex indicates a unique tree dimension. (A) The tree is two dimensional with *T*_*d*_=2, *D*_*m*_=6 and *L*=4. (B) The tree is four dimensional with *T*_*d*_=2.28, *D*_*m*_=6 and *L*=8.

Tree dimension measure *T*_*d*_ exploits the benefits of tree diameter *D*_*m*_ while mitigating its shortcomings by incorporating the number of leaves *L*. Fig 3 illustrates the fundamental differences of *T*_*d*_ that results in better performance than those of *D*_*m*_ and *L*. Fig 3A shows a multifurcating tree with *T*_*d*_ = 2, *D*_*m*_ = 6 and *L* = 4. Fig 3B shows a tree with *T*_*d*_ = 2.28, *D*_*m*_ = 6 and *L*=8. Both trees have the same *D*_*m*_. The tree in Fig 3B has more short branches but only inflating its *T*_*d*_ value slightly. *D*_*m*_ is the same for both trees, insensitive to the structural differences. This underscores the insensitivity of *D*_*m*_ to secondary branching, while *T*_*d*_ captures both global and local tree properties. The value of *L* on the tree in Fig 3B drastically doubles to 8, highlighting a case where *L* can be locally extreme. The superior performance of *T*_*d*_ over *D*_*m*_ and *L* is further demonstrated in *Results*.

### The tree dimension test for trajectory presence

We now describe the tree dimension test (TDT), a statistical test for the presence of trajectory. Tree dimension measure *T*_*d*_ is a useful mathematical descriptor of trajectory pattern, though not fully statistically empowering. Thus we introduce trajectory test statistic *S*:

$$\begin{array}{c} \text{Test}\;\text{statistic}\;S=N\cdot \frac{\text{log}\;\text{max}\left(T_{d}\right)-\text{log}T_{d}}{\text{log}\;\text{max}\left(T_{d}\right)-\text{log}\;\text{min}\left(T_{d}\right)} \end{array}$$ (2)

where *N* is the number of vertices in the tree, and the minimum and maximum of *T*_*d*_ are given by Propositions 1 and 2. *S* is negatively associated with *T*_*d*_. A strong trajectory pattern will have a large value of *S*. Most importantly, *S* increases with sample size *N* to promote statistical support not reflected in *T*_*d*_.

We also define the test effect size by *S*/*N* that is the log of tree dimension measure *T*_*d*_ negatively and linearly scaled to [0, 1], with 0 depicting no trajectory patterns and 1 a perfect trajectory pattern.

To test trajectory presence, we define the null hypothesis that no trajectory patterns are present in the data. We choose the null population to follow a spherical multivariate normal (MVN) distribution, because it presents an isotropic point cloud that attenuates from the mean, representing a pattern one would not expect a trajectory.

Next we obtain the null distribution of test statistic *S*. Given an input dataset with *N* samples and *d* dimensions, we obtain *N* samples of *d*-dimensional random vectors from a spherical MVN distribution with an identity covariance matrix. We compute one *S* on these samples. Repeating the procedure many times, we obtain the empirical null distribution of *S* (Alg. 2 Null-Distribution).

Our experimental results suggest that the null distribution of *S* can be best approximated by a log-normal distribution based on the Bayesian Information Criterion (BIC) and Akaike Information Criterion (AIC) for model selection. We also performed the Kolmogorov–Smirnov (KS) test to determine equality of the *S* statistic null distribution to three candidate distributions: log-normal, gamma, and normal. Each was fit to the empirical null distribution of *S* obtained from 1000 samples of data from an eight dimensional spherical MVN distribution. The two parameters of each distribution were obtained by maximum likelihood estimation.

**Fig 4:**
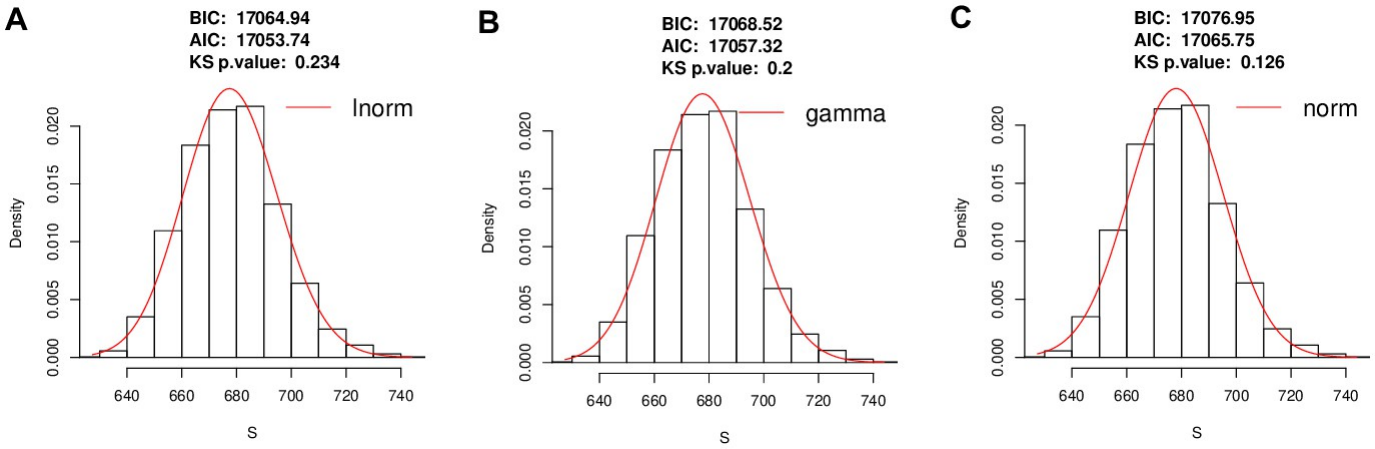
Approximating the empirical null distribution of trajectory test statistic *S*. BIC, AIC, and KS test *p*-values of (A) log-normal (lnorm), (B) gamma, and (C) normal (norm) distributions after they (red curves) were fit to simulated null test statistic *S* values (histograms).

The log-normal distribution, achieving the lowest (best) BIC and AIC scores (Fig 4), and the least significant deviation from the data by the KS test *p*-value, is considered the best option for the null distribution of *S*. Using the upper-tail probability of the null distribution, we can compute the statistical significance (*p*-value) of an observed test statistic *S* (Alg. 3 Test-Trajectory-Presence). A decision on trajectory presence can be thus made at a given type I error rate (e.g., 0.05).

**Algorithm 2** Null-Distribution(*X*, *B*)

 Input: *X* is multivariate data of *N* samples in *d* dimension;

      *B* is the number of random samples for the null population

1 **for** *i* = 1 **to** *B*

2    // Get *N* samples of *d* dimensional spherical MVN random

      vector by Monte Carlo sampling:

3    *X*_*π*_ = Spherical-MVN(*N*, *d*)

4    *T*_*d*_ = Tree-Dimension-Measure(*X*_*π*_)

5    *S*_0_[*i*] = Tree-Dimension-Statistic(*T*_*d*_)

6 Fit a log-normal null distribution by maximum likelihood estimation

$\left(\hat{\mu },{\hat{\sigma }}^{2}\right)={\text{argmax}}_{\mu ,\sigma^{2}}L\left(\mu ,\sigma^{2}|S_{0}\right)$

7 **return** log-normal null probability density function $f_{0}\left(s|\hat{\mu },{\hat{\sigma }}^{2}\right)$

### Time complexity of tree dimension test for trajectory presence

We now analyze the runtime of the tree dimension algorithms. Given multivariate data *X* with *d* dimensions and sample size *N*, Alg. 1 Tree-dimension computes an EMST in approximately *O*(*N* log *N*), using the dual-tree Boruvka algorithm [11] as implemented in the ‘mlpack’ software package [12]. For Alg. 2 Null-Distribution with *B* simulations, the runtime is *O*(*BN* log *N*). Thus, the overall runtime complexity is approximately *O*(*BN* log *N*) for Alg. 3 Test-Trajectory-Presence.

### Measuring the tissue specificity to gene expression dynamics by minimum subtree cover

Given observed gene expression data from multiple tissue types, we examine whether data points from one tissue type are close to each other in gene expression or mixed with other tissue types. This allows one to assess tissue specificity. If all genes in a genome are included in the expression data, the corresponding tissue specificity is regarding the cellular states involving all genes for a tissue type; if only genes in a pathway are examined, the tissue specificity is regarding the dynamics of a pathway for a tissue type. Here we study tissue specificity using the EMST *T* of given gene expression data.

**Algorithm 3** Test-Trajectory-Presence(*X*)

 Input: multivariate data *X*

1 Null distribution $f_{0}\left(s|\hat{\mu },{\hat{\sigma }}^{2}\right)$ = Null-Distribution(*X*, *B*)

2 Tree dimension measure *T*_*d*_ = Tree-Dimension-Measure(*X*)

3 *S* = Tree-Dimension-Statistic(*T*_*d*_)

4 $p\text{-value}=\int_{S}^{\infty }f_{0}\left(s|\hat{\mu },{\hat{\sigma }}^{2}\right)ds$

5 **return** *p*-value

We now define a tissue specificity measure on *T* with a vertex set *V*, where each vertex is labeled with a tissue type. Let *W*_*t*_ be the subset of vertices labeled by tissue type *t* in *V*. The key step is to find a subtree *T*_*t*_ of *T* such that *T*_*t*_ covers *W*_*t*_ with the minimum number of vertices. Let *V*_*t*_ be the vertex set of *T*_*t*_, which may contain vertices not labeled by *t*.

We define the specificity of tree *T* to tissue *t* by

$$\begin{array}{c} \theta \left(t\right)=\frac{|W_{t}|}{|V_{t}|} \end{array}$$ (3)

where the absolute value of a set is its cardinality. If the minimum subtree cover has all its vertices labeled by the same tissue type *t*, then the tissue specificity for tissue *t* is 1. Tissue specificity decreases when the minimum subtree cover contains many vertices that do not belong to tissue type *t*. We use $\bar{\theta }$ to denote tissue specificity of a tree by the average tissue specificity to each of *M* tissue types:

$$\begin{array}{c} \bar{\theta }=\frac{1}{M}\sum_{t=1}^{M}\theta \left(t\right) \end{array}$$ (4)

To identify the cover vertex set *V*_*t*_ for vertex subset *W*_*t*_, we introduce Algorithm 4 Minimum-Subtree-Cover that obtains a minimum subtree *T*_*c*_ with vertex set *T*_*c*_ and edge set *E*_*c*_ that covers a vertex subset *W* in a tree *T*. Algorithm 5 Cover-DFS finds all vertices to be included in the minimum subtree cover using DFS traversal. The first vertex to be explored by Cover-DFS must be in vertex subset *W*. During DFS traversal, if a vertex *u* is in the minimum subtree cover, its discovery vertex *v* must be included in the cover.

**Algorithm 4** Minimum-Subtree-Cover(tree *T*, vertex subset *W*)

 Input: *T*, a tree (*V*, *E*) with vertex set *V* and edge set *E*

      *W*, a subset of vertex set *V* to find a minimum subtree cover

1 Let *w* be a vertex in subset *W*

2 Cover-DFS(*T*, *W*, *w*) // To identify vertices in the subtree cover

3 Minimum subtree cover vertex set *V*_*c*_ = {*v* | *inCover*[*v*] = true, *v* ∈ *V*}

4 Minimum subtree cover edge set *E*_*c*_ = {(*u*, *v*) | *u*, *v* ∈ *V*_*c*_, (*u*, *v*) ∈ *E*}

5 **return** minimum subtree *T*_*c*_ = (*V*_*c*_, *E*_*c*_) that covers all vertices in *W*

We now analyze the runtime of Alg. 4 Minimum-Subtree-Cover. For an input tree with *N* vertices and *N* − 1 edges, the running time complexity of Cover-DFS on the tree is *O*(*N* + (*N* − 1)) = *O*(*N*). Tracing out the minimum subtree also takes *O*(*N* + (*N* − 1)). Therefore, the overall running time complexity for finding the minimum subtree cover of a given subset of vertices is *O*(*N*), linear to the number of vertices in the input tree.

**Fig 5:**
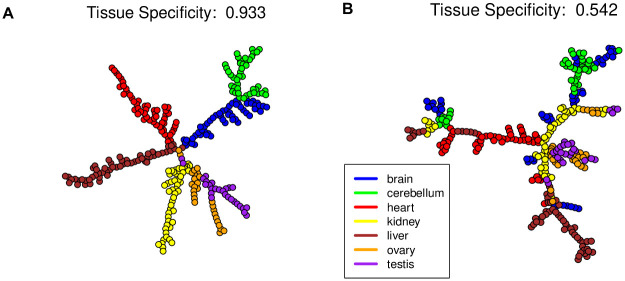
Two pathways of contrasting tissue specificity during the development of seven mouse tissues. EMSTs linking tissue samples are derived from expression levels of genes on each pathway. Vertices are samples colored by tissue type. (A) The Wnt signaling pathway is of high tissue specificity in gene expression dynamics, with developing samples of the same tissue type forming unique trajectory segments. (B) The mismatch repair pathway, of low tissue specificity in gene expression dynamics, shows mingled samples of different tissue types.

Tissue specificity to gene expression of two pathways in seven developing mouse tissues are illustrated in Fig 5. The Wnt signaling pathway has a high tissue specificity of 0.933, where the different tissue samples are well separated in gene expression (Fig 5A). The mismatch repair pathway has a low tissue specificity of 0.542 (Fig 5B).

**Algorithm 5** Cover-DFS(tree *T*, vertex subset *W*, vertex *v*)

 Input: *T*, a tree (*V*, *E*) with vertex set *V* and edge set *E*

      *W*, a subset of vertex set *V* to find a sub-tree cover

      *v*, a vertex in *V* to be explored

1 Mark *v* as visited

2 **if** vertex *v* is a member of subset *W*

3  *inCover*[*v*] = true

4 **else**

5  *inCover*[*v*] = false

6 **for** each vertex *u* adjacent to *v* in tree *T*

7  **if** *u* is not visited

8   Cover-DFS(*T*, *W*, *u*)

9   **if** *inCover*[*u*] is true

10    *inCover*[*v*] = true

## Results

### Performance evaluation of tree dimension test on simulated data

We first evaluated the performance of Alg 3. Test-Trajectory-Presence(*X*) in recognizing trajectory patterns against the more intuitive alternatives the number of leaves *L* and tree diameter *D*_*m*_ on synthetic data, which includes 229 simulated single-cell datasets from the ‘Dynverse’ trajectory inference project [13]. These datasets have various types of trajectories: bifurcating, multifurcating, tree, and cycle, constituting ground-truth trajectories; we shuffled each dataset to generate another 229 datasets that represent ground-truth examples of no trajectories.

To preprocess the data, we perform dimensionality reduction using principal component analysis (PCA). Most trajectory inference methods employ dimensionality reduction before finding a trajectory. PCA may potentially not preserve trajectory but it captures variations that constitute the primary dynamics in the data. We employed the CNG scree test to select the best number of principal components [14, 15]. The TDT method is not tied to PCA and other data preprocessing procedure could be used as reflected in the design of our accompanying software.

**Fig 6:**
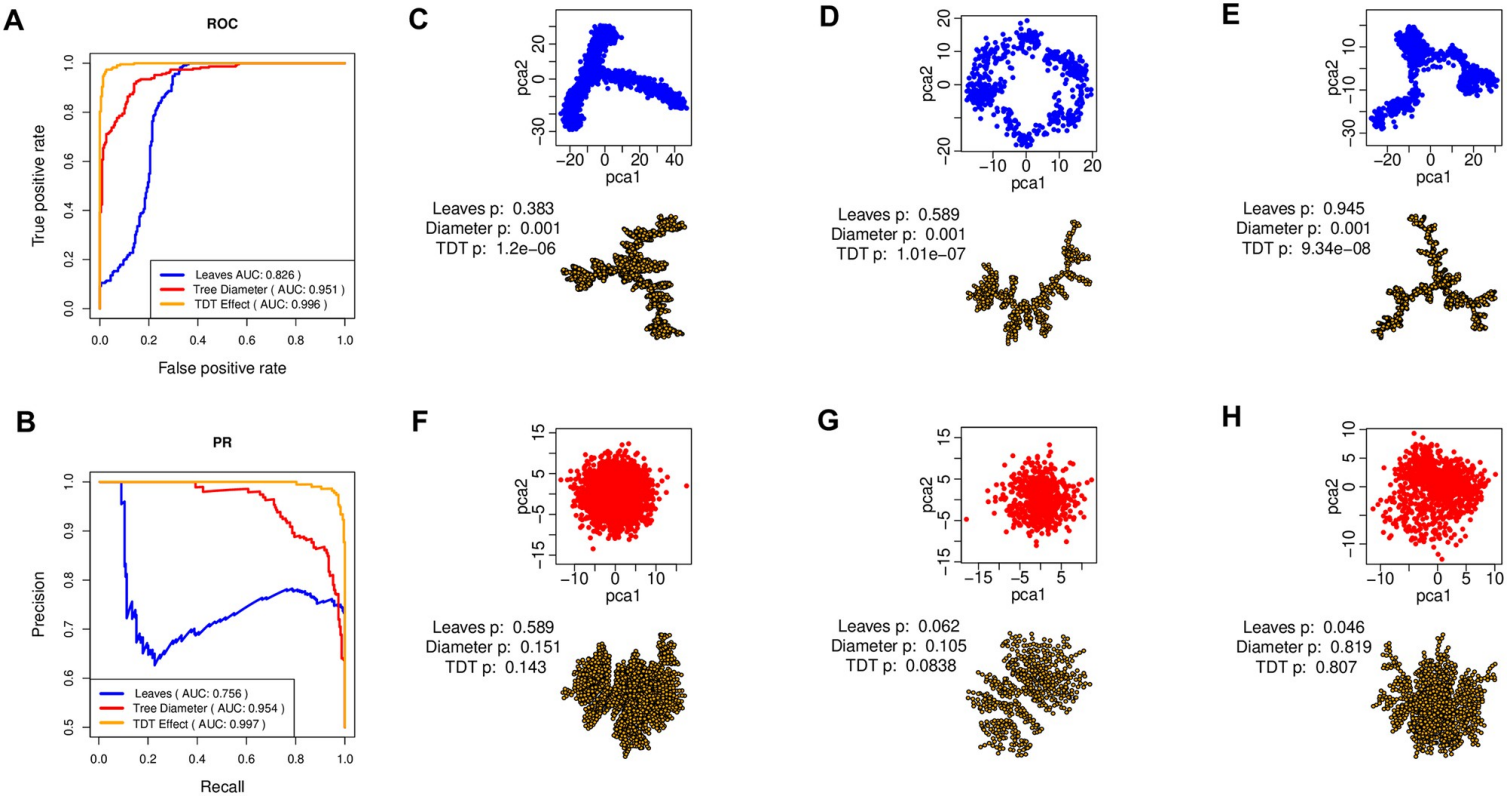
Trajectory presence testing on 229 simulated single-cell datasets. (A) ROC curves and AUROC scores for TDT effect size *S*/*N*, number of leaves *L* and tree diameter *D*_*m*_. (B) PR curves and AUPR scores for the three methods. (C) The PCA plot of a multifurcating trajectory patterns, points represent cell and the axes gene expression, and the EMST representation of trajectory pattern, with each point representing a cell. Text shows *p*-values of each method when applied on the dataset. (D) PCA and EMST plots of a looping trajectory pattern. (E) PCA and EMST plots of a multifurcating trajectory. (F)–(H) PCA and EMST plots of datasets with no significant trajectory patterns.

Each method was applied to produce 458 scores, one for each dataset. We use normalized scores of 1 − *L*/*N* and *D*_*m*_/*N* for number of leaves and tree diameter, respectively. The TDT effect size *S*/*N* is used as score for tree dimension test. The normalization is employed to make datasets of different sample sizes comparable for performance evaluation. The scores are then used to plot receiver operating characteristic (ROC) and precision-recall (PR) curves and calculate area under the ROC curve (AUROC) and area under the PR curve (AUPR) for each method as its performance (Fig 6). In Fig 6A, TDT has the best AUROC score of 0.996, while *D*_*m*_ has an AUROC score of 0.951, and *L* has the least AUROC score of 0.826. Similarly, Fig 6B shows PR curves and the AUPR scores of the methods. TDT has the highest AUPR score of 0.997, while *D*_*m*_ has a score of 0.954, and *L* has a score of 0.756. The high AUC scores of TDT demonstrate the effectiveness of tree dimension measure in recognizing trajectory-like from isotropic patterns over other more intuitive methods.

Fig 6C–6E display different types of trajectory patterns used in the experiment, as well as the statistical significance for the presence of trajectory as represented by the *p*-values of each method. Each dataset is summarized by a principal component analysis (PCA) plot and the corresponding EMST representations. The EMST representation preserves the structure in data, that is the spatial relationships between data points. We set the edges in the EMST to length one to capture the topology. This EMST representation is sufficient for the subsequent step of recognizing presence of a trajectory. Fig 6C presents simulated data with trajectory and TDT correctly recognized the presence of a trajectory in the data as denoted by the significant *p*-value of 1.2e-06. Similarly, TDT also recognizes the presence of trajectory in Fig 6D with a *p*-value of 1.01e-07, and the trajectory in Fig 6E with a *p*-value of 9.34e-08. Number of leaves *L* failed to recognize these patterns as denoted by the relatively high *p*-values. Tree diameter *D*_*m*_, however, can recognize the trajectory patterns as indicated by the significant *p*-values. Fig 6F–6H give examples of patterns without a trajectory. All methods correctly recognized the absence of trajectory patterns here as denoted by the relatively high and less significant *p*-values.

### Performance evaluation of tree dimension test on single-cell data

**Fig 7:**
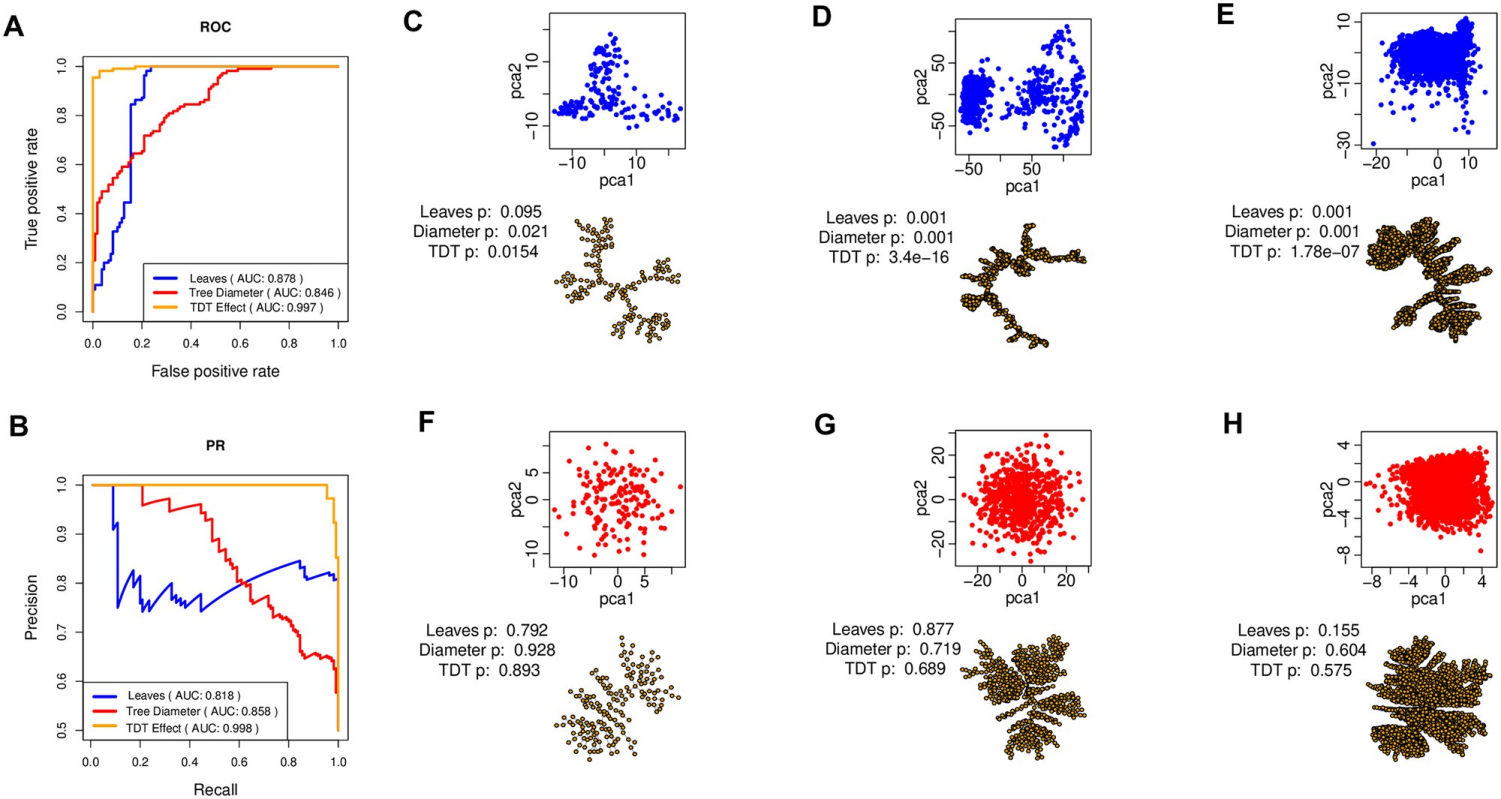
Trajectory presence testing on 110 real single-cell datasets. (A) ROC curves and AUC scores for TDT effect size *S*/*N*, number of leaves *L* and tree diameter *D*_*m*_. (B) PR curves and AUC scores for the three methods. (C) The PCA plot and EMST representation of trajectory patterns in single-cell human lung adenocarcinoma cancer cell lines data. Points represent cells. Text shows *p*-values of each method when applied on the dataset. (D) PCA and EMST plots of a trajectory pattern in human female germline single-cell data (E) PCA and EMST plots of a trajectory pattern in planaria single-cell data. (F)–(H) PCA and EMST plots of datasets with no significant trajectory patterns.

We evaluated the three methods on 110 curated real single-cell datasets known to have trajectories, also used in the ‘Dynverse’ project [13]. They constitute the ground-truth patterns with trajectories. To obtain the controls, we shuffled the genes to make them statistically independent to generate another 110 datasets of no trajectory patterns in the ground-truth. Fig 7 shows the results. Fig 7A shows ROC curves and the corresponding AUROC scores for each method. The TDT effect size has the best performance with an AUROC score of 0.997, while *L* has an AUROC score of 0.878 and *D*_*m*_ has the lowest AUROC score of 0.846. Similarly, Fig 7B shows PR curves, with the TDT effect size having the best AUPR of 0.998. *L* has the least AUPR score of 0.818 and *D*_*m*_ has an AUPR score of 0.858. These results demonstrate the effectiveness and robustness of the tree dimension test in recognizing trajectory from random patterns in real single-cell data, which are noisy and subject to the dropout effect.

Fig 7C–7E show true trajectory patterns as represented by the PCA plots and EMST representations. Fig 7C is human lung adenocarcinoma cell line data with trajectory. Fig 7D is human female germline single-cell data [16] with trajectory. Fig 7E is planaria data with trajectory. All the methods are able to recognize the presence of trajectory patterns in these datasets as denoted by the significant *p*-values, except the lung cancer data (Fig 7C) where the *p*-value of *L* is insignificant. Fig 7E highlights the limitation of using only two-dimensional PCA to visualize patterns in the data, while the EMST representation shows interesting dynamical patterns in data. Fig 7F–7H show patterns without trajectory. No method statistically validates the presence of trajectories in these datasets as denoted by the relatively high *p*-values.

### Empirical runtime of tree dimension test on simulated large datasets

**Fig 8:**
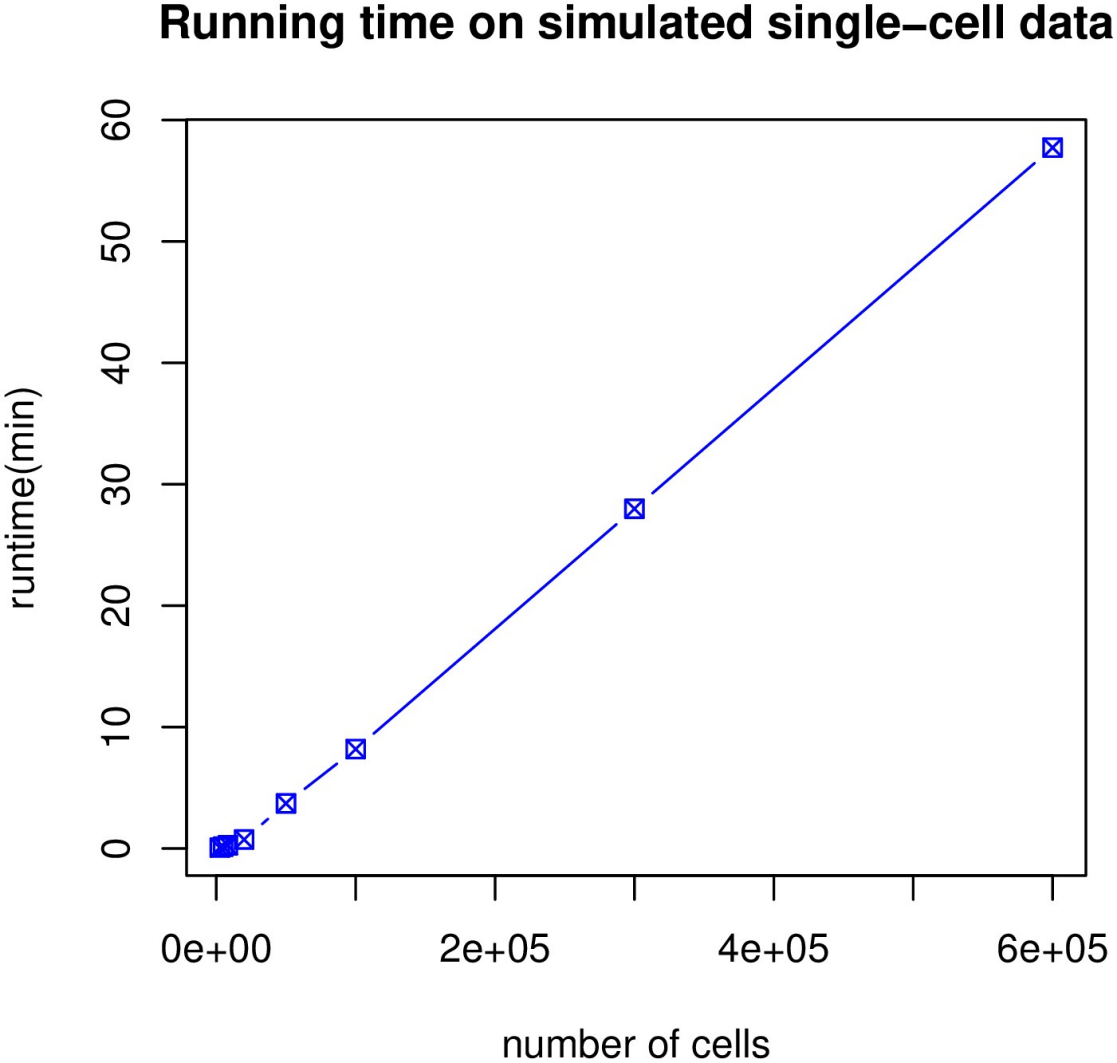
Empirical runtime of TDT on simulated single-cell data with varying number of cells. The runtime includes 100 simulations to estimate parameters for the null distribution. The horizontal axis represents the number of cells. The vertical axis is the runtime in minutes. Time was recorded on a 2015 Apple Macbook Pro laptop computer with 2.2 GHz Quad-Core Intel Core i7 processor using a single thread.

A large single-cell sequencing dataset can contain a million cells (*N* = 10^6^). An exact EMST solution taking quadratic time *O*(*N*^2^) is not practical. So our study uses the established dual-tree Boruvka algorithm [11] to find approximate EMSTs in time complexity *O*(*N* log *N*). Fig 8 shows the empirical runtime of TDT on simulated data with varying numbers of cells using the approximate Boruvka algorithm. This runtime includes 100 simulations to establish null distribution parameters, which could be precomputed. The results show that the practical runtime scales about linearly to *N*. The efficiency on hundreds of thousands of cells is reasonable as one would expect for a single-cell analysis.

Furthermore, the TDT effect size for trajectory presence is calculated on the input data without simulations, thus computationally much faster (in about 1% of the shown time) to obtain than the *p*-value. In real applications, it may be necessary to only compute *p*-values for data with a reasonably strong effect.

### Pathways exhibit diverse trajectory patterns distinct from cellular trajectories during development

**Fig 9:**
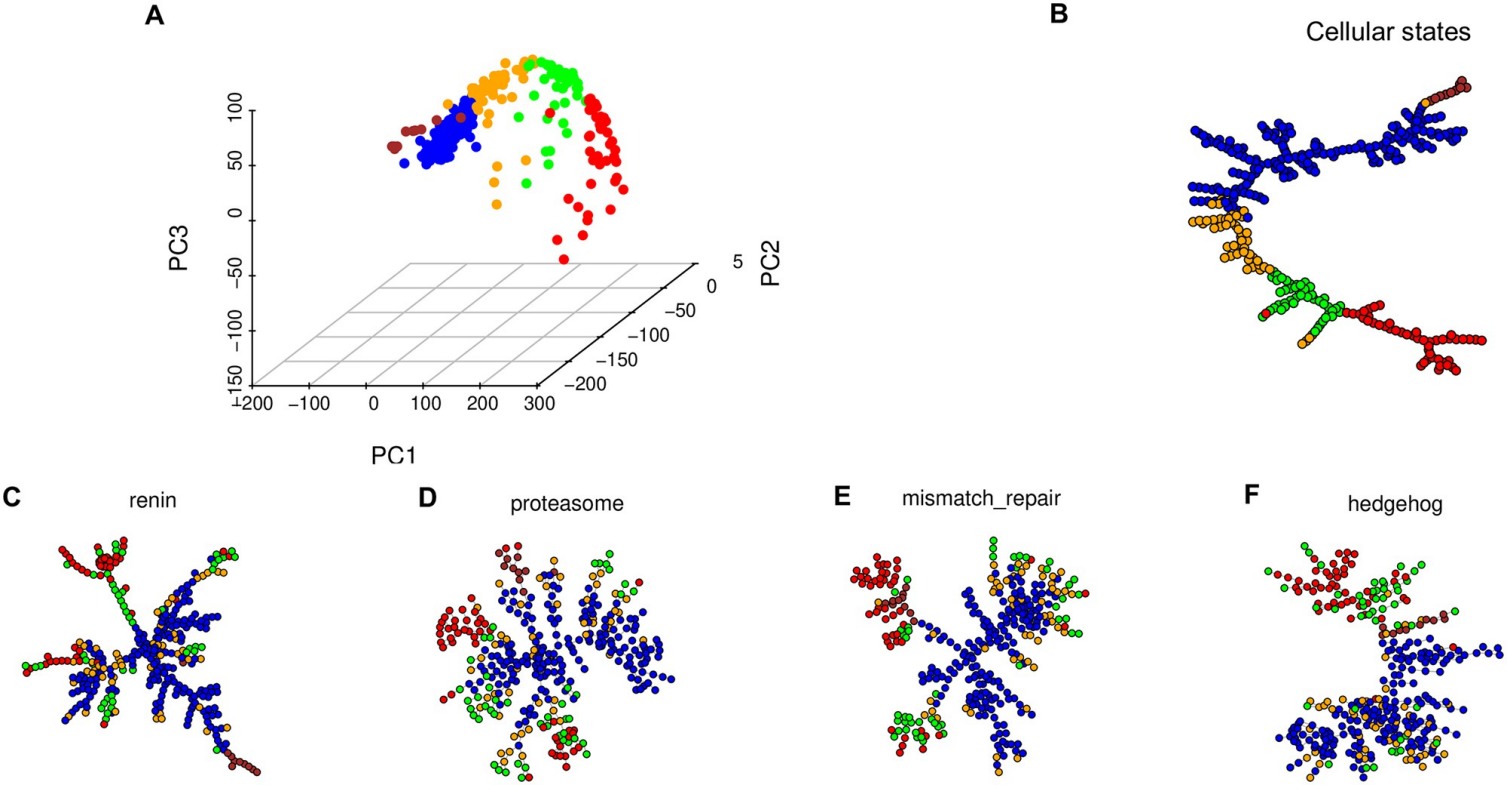
Distinct cellular and pathway trajectories in single-cell data of embryonic stem cells. Cells are clustered into five groups indicated by the colors. (A) Observed gene expression data of embryonic stem cells in the first three principal components. (B) The EMST of the entire transcriptome suggests a strong trajectory pattern at the cellular level. (C)-(F) EMST representations of trajectory patterns in embryonic stem cells using gene expression on the pathways of (C) renin, (D) proteasome, (E) mismatch repair, and (F) hedgehog.

The ability to test the presence of trajectory patterns opens up the possibility of examining pathway dynamics beyond cellular dynamics. A pathway trajectory is spanned by genes in that pathway. We examined whether pathway trajectories may be dynamically different from cellular trajectories in a single-cell dataset of mouse embryonic stem cells [17]. Fig 9A is the PCA plot of the cells clustered into five groups. Fig 9B is the EMST representation of the cells showing a strong trajectory pattern with a significant *p*-value by tree dimension. Fig 9C–9F are EMST representations of the gene expression dynamics of four pathways. These plots highlight structural differences of the EMST representations between cellular and pathway trajectories. The color coding shows that the spatial relationships of the cells are preserved neither between the cellular and pathway EMSTs nor among the pathway EMSTs. This demonstrates that pathway trajectory patterns can indeed be distinct from cellular trajectory patterns.

### Tissue specificity of gene expression dynamics varies markedly over pathways during mammalian development

Now we characterize pathway dynamics by tissue specificity. A pathway is tissue specific if expression of genes in the pathway are homogeneous among samples from the same tissue type, but heterogeneous across samples from other tissue types. As a result, a pathway is tissue specific if the samples from that tissue are in the same neighbourhood in the EMST representation of trajectory patterns. We exploit this property to quantify the average tissue specificity of a pathway across all tissue types in a dataset. We examined a mammalian developmental transcriptome collection covering seven tissue types from embryonic stages to adulthood in human and mouse [18], including cerebrum, cerebellum, heart, kidney, liver, ovary and testis.

**Fig 10:**
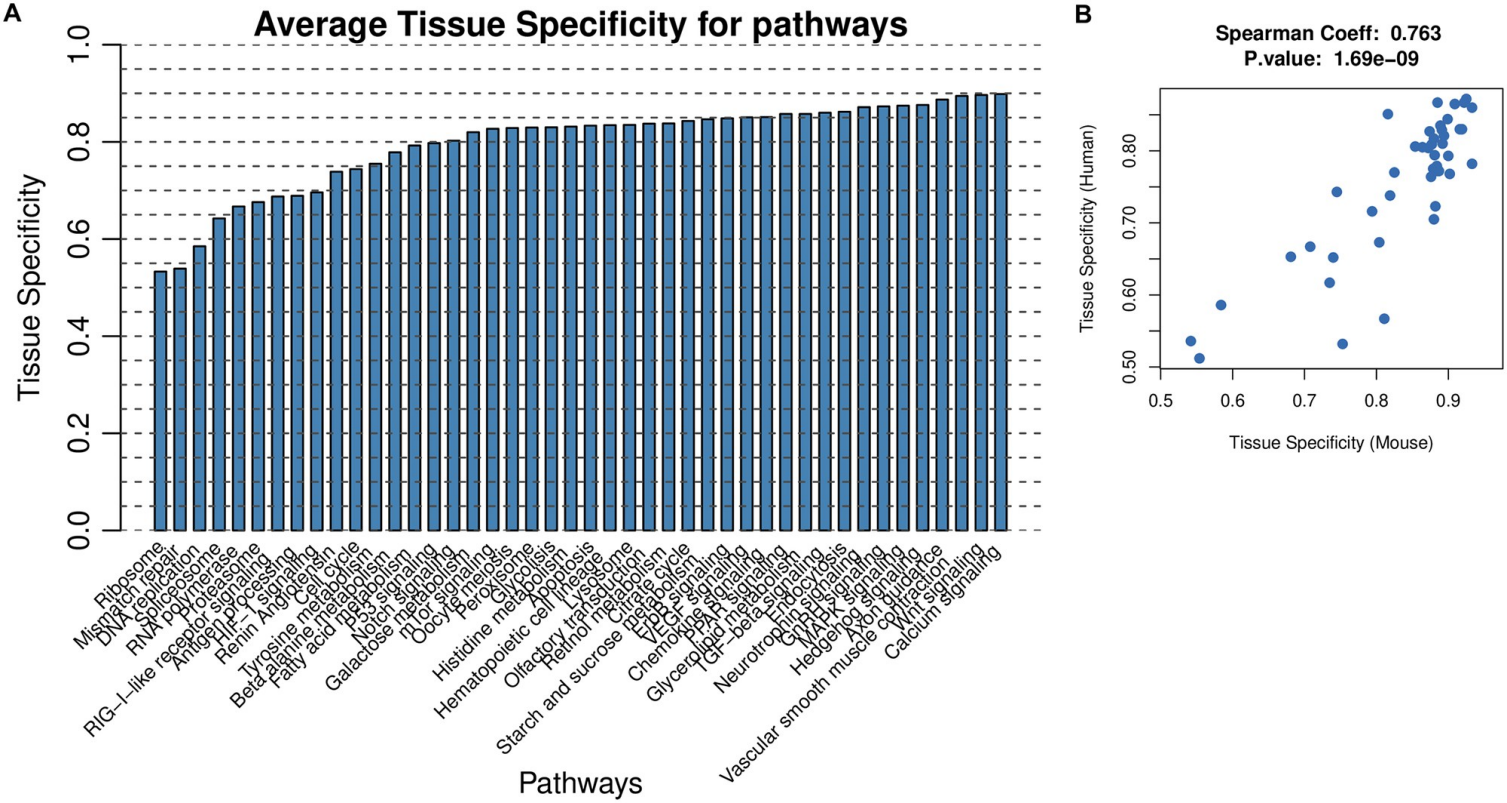
Tissue specificity of gene expression dynamics of 40 biological pathways during human and mouse development. (A) Pathways are ranked by average tissue specificity during mammalian development computed from transcriptome data in seven tissue types in human and mouse. (B) Pathway tissue specificity is conserved between developing mouse and human. Each point is a pathway and the axes represent its tissue specificity scores for mouse and human, respectively.

We tested the presence of trajectory patterns and measured the tissue specificity for 40 KEGG pathways. Fig 10A ranked the pathways by average tissue specificity during mouse and human development. Calcium signaling is the most tissue-specific pathway with an average tissue specificity of 0.899. The ribosome pathway is least tissue specific with an average tissue specificity of 0.533. Fig 10B visualizes how pathway tissue specificity is conserved between human and mouse. Tissue specificity of the 40 pathways is highly correlated between human and mouse with a Spearman’s rank correlation coefficient of 0.763 (*p*-value = 1.69 × 10^−9^).

### Pathways with most and least tissue-specific expression dynamics

**Fig 11:**
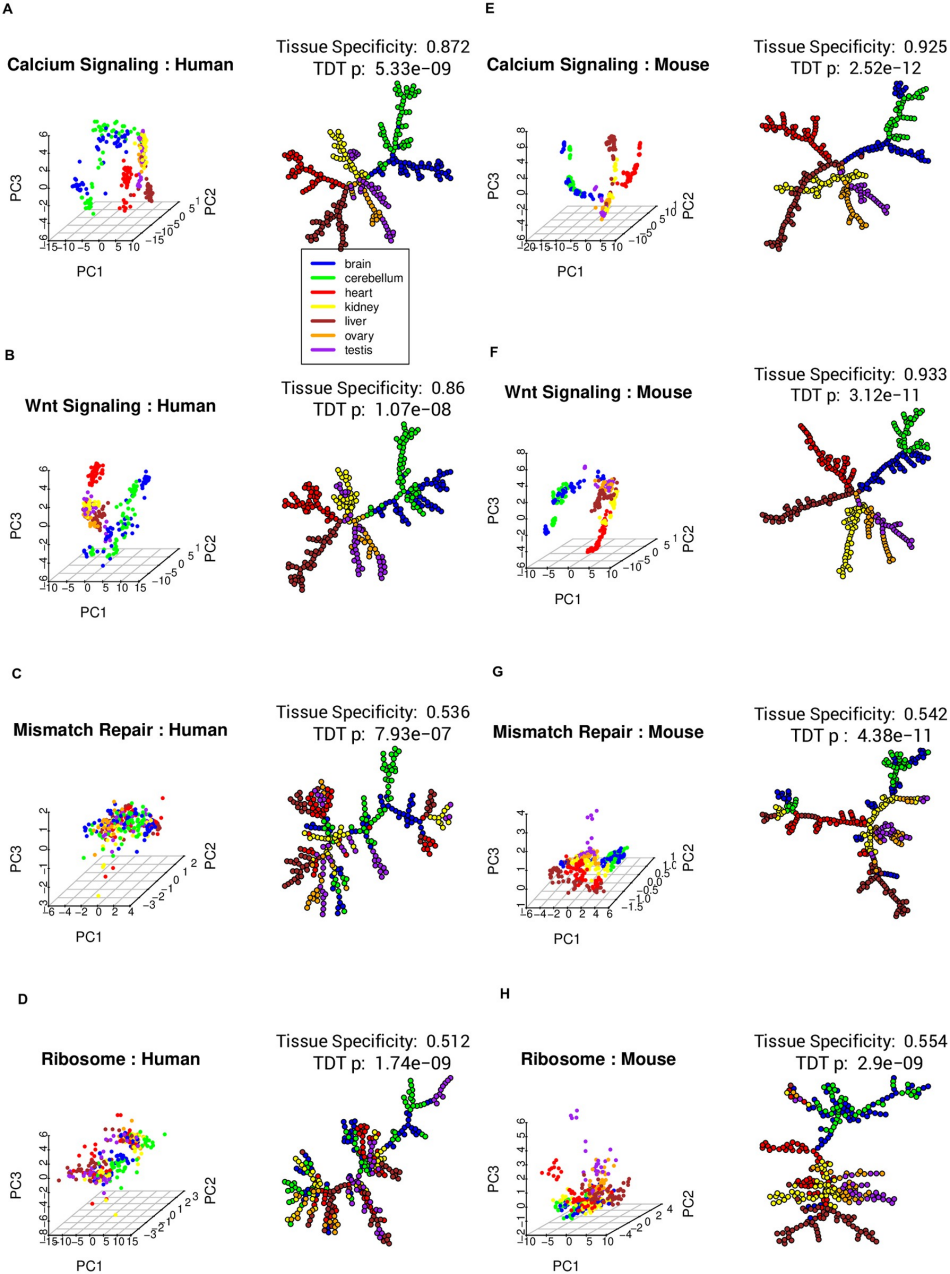
Gene expression dynamics of most and least tissue-specific pathways during mammalian development. Seven tissue types are coded by colors. PCA and EMSTs of expression of genes on (A) calcium signaling, (B) Wnt signaling, (C) mismatch repair, and (D) ribosome pathways during human development. (E)–(H) PCA and EMSTs of gene expression dynamics of the same four pathways during mouse development.

Fig 11 shows gene expression dynamics of two most and two least tissue-specific pathways during human and mouse development, respectively. Both Wnt and calcium signaling pathways present strong trajectory patterns that are also highly tissue specific as indicated by trajectory segments covering unique tissue types; the mismatch repair and ribosome pathways present weak trajectory patterns while exhibiting relatively low tissue specificity.

Pathway tissue specificity is the degree of homogeneity of pathway gene expression dynamics among samples from the same tissue type. In Fig 11, calcium signaling and Wnt signaling pathways are highly tissue specific during mammalian development: samples from the same tissue type are close together in the EMST representation of the trajectory patterns. Most highly tissue-specific pathways are involved in signal transduction.

Calcium signaling, important throughout development, regulates cellular processes such as division, migration, death, and differentiation [19]. Its mechanisms are unique in regulating the calcium oscillation frequency in different tissue types. Studies have identified multiple isoforms of calcium receptors that are specific to tissue types [20]. Since these receptors control the transportation of calcium ions into cells, multiple isoforms of receptors result in unique downstream calcium signals in different tissues. For example, calcium signaling has been observed to exhibit tissue specificity in mitochondrial calcium uptake [21].

Wnt signaling is known to be universal and essential for development. As such, it has long been presumed that the pathway results in uniform responses from different tissue types. However, emerging evidence suggests that the pathway drives unique responses in distinct tissue types, and is conjectured to be tissue specific [22]. For example, the Wnt signaling pathway was found to have unique susceptibility loci for systolic blood pressure [23].

Mismatch repair and ribosome pathways are low in tissue specificity during development (Fig 11). Many such pathways are involved in genetic information processing.

Our findings revealed high tissue specificity of several signal transduction pathways, but low tissue specificity in genetic information processing pathways, as partially supported by limited literature. Further biological inquiries are needed to either support or counter our findings regarding pathway tissue specificity, a direction still under-explored.

## Discussion

### Tree dimension as a favorable statistic for the strength of a trajectory pattern

The EMST representation of multivariate data is both computationally efficient and globally optimal, widely used by trajectory inference methods such as Slingshot [1]. Our premise is that the presence of dynamical patterns in data can be characterized by the degree of linearity of its corresponding EMST. All three EMST statistics we have evaluated attempt to characterize the degree of linearity of an EMST and in effect, presence of trajectory patterns. All are able to differentiate between extreme cases of linearity. Tree dimension measure *T*_*d*_ and the number of leaves *L* are minimized if the EMST is linear and maximized if it is a star tree; tree diameter *D*_*m*_ is maximized by a linear EMST and minimized by a star tree. They differ, however, in the global and local details they capture. On the one hand, the number of leaves *L*, driven by local details, would not differentiate between a linear trajectory and a bifurcating trajectory with the same number of leaves as noisy states. On the other hand, tree diameter *D*_*m*_, driven by the global longest path, is unable to differentiate between a tree with few long branches and a tree with many short branches if both have the same longest path length. The tree dimension measure is designed to capture directionality/linearity of trees. It offers a more balanced measure of linearity, integrating the more intuitive *L* and *D*_*m*_, which can be susceptible to local and global extremes, respectively. *T*_*d*_ attempts to combine the strengths of *D*_*m*_ and *L* while simultaneously mitigating their limitations. This gives tree dimension an edge in performance over the other alternatives as we have demonstrated in simulation studies.

### The use of Euclidean minimum spanning trees

Our choice of EMST is informed by a number of considerations. First, EMST is a globally optimal compact structure to summarize multivariate data in the Euclidean space. Second, it has no tunable parameter to change the representation dramatically on the same data. Third, it is widely tested in trajectory inference applications: some of the most popular trajectory inference methods such as TSCAN [5] and Monocle [24] employ MST with clustering to capture trajectories in data.

Notably, for a specific application where EMST is deemed not relevant, our method is still applicable to any other type of tree summary of a dataset, or a tree summary resulting from a graph representation of a dataset. The context of trajectory testing remains valid as long as vertices in a tree are derived according to some defined proximity measure.

Finding MSTs on clusters of cells is a promising direction. Even though efficient, the performance for trajectory presence testing based on our early unpublished work is not as good as we have presented here. The performance depends on cluster shapes and the number of clusters. Popular clustering methods seem to be ineffective in our context, implying a need to develop trajectory-friendly clustering strategies.

### Between spherical multivariate normal distribution and perfect linear trajectory

We use spherical MVN distribution to represent data for generating the null distribution for the tree dimension test statistic. Spherical MVN represents isotropic patterns that do not have any orientation or direction, thus no trajectory. The other extreme is a perfect linear pattern where the EMST is a path graph, corresponding to a one dimensional manifold. Although TDT is designed to prioritize trajectories with intrinsic dimensionality of one, it has the capability to capture cases on a spectrum from highly linear trajectory to isotropic patterns as represented by the spherical MVN distribution. Therefore, our method may accept trajectory presence if data reside in a subspace with lower intrinsic dimensionality other than one, out of a much higher-dimensional space. Even though the dimensionality may be greater than one, the pattern can be much closer to a trajectory than being isotropic.

### The null distribution of tree dimension test statistic

The parameters (mean and standard deviation) of log-normal null distribution of tree-dimension statistic *S* depend on sample size and the number of dimensions of the dataset. Our approach samples from the null population to obtain maximum likelihood estimates of the parameters. The sampling is computationally intensive if the input dataset is of large sample size and high dimensionality, as is often the case with single-cell data. Possible future work is to avoid sampling by deriving a theoretical asymptotic null distribution for the test statistic *S*.

### Pathway trajectories may not follow a cellular trajectory

Since a pathway space is spanned by a subset of genes, it is evident that the dynamics of a point cloud from a pathway may be different from the one that is spanned by all genes. Therefore, pathways may reveal strong dynamical patterns not present if the whole gene set is used. It is also possible that not all pathways result in strong trajectory patterns.

In Fig 9, pathway dynamics are different from the global dynamics of all genes. Moreover, the color coded points by tissue types highlight a difference in spatial distribution between different pathways. Points concentrated in one neighborhood in the subspace of expression dynamics of one pathway may be scattered in the subspace of expression dynamics of another pathway, giving rise to diverse patterns.

## Conclusion

We have developed the tree dimension test to infer trajectory presence in multivariate data. Our studies on both simulated and biological data validated the effectiveness of the method over other options. The methodology enables pathway prioritization by trajectory strength beyond the overall cellular dynamics. By the presented sub-tree cover method, we further discovered striking differences regarding the tissue specificity of pathway dynamics during mammalian development: several signal transduction pathways are highly tissue specific in gene expression dynamics, while genetic information processing pathways tend to be low in tissue specificity. Our work complements existing trajectory inference methods in providing statistical support for the significance of a potential trajectory pattern, also opening a window to prioritize pathways by dynamics to uncover detailed molecular mechanisms of a biological system.
